# COMMD4 is a novel prognostic biomarker and relates to potential drug resistance mechanism in glioma

**DOI:** 10.3389/fphar.2022.974107

**Published:** 2022-09-30

**Authors:** Zongheng Liu, Long Peng, Yidan Sun, Zhichao Lu, Bing Wu, Weichen Wang, Xiaomei Zhang, Haiyan Hao, Peipei Gong

**Affiliations:** ^1^ Department of Neurosurgery, Affiliated Hospital of Nantong University, Medical School of Nantong University, Nantong, China; ^2^ Postgraduate School, Dalian Medical University, Dalian, China; ^3^ Department of Oncology, First Teaching Hospital of Tianjin University of Traditional Chinese Medicine, Tianjin, China; ^4^ Department of Outpatient, Affiliated Hospital of Nantong University, Nantong, China

**Keywords:** CGGA, TCGA, COMMD4, glioma, drug resistance, mast cells

## Abstract

**Background:** Glioma as the most frequently discovered tumor affecting the brain shows significant morbidity and fatality rates with unfavorable prognosis. There is an urgent need to find novel therapeutic targets to overcome the low chemotherapeutic efficacy of glioma. This research examined whether the copper-metabolism-domain protein, COMMD4, had predictive and therapeutic significance in glioma.

**Methods:** Using the freely accessible CGGA (The Chinese Glioma Atlas) and TCGA (The Cancer Genome Atlas) databases, we examined the function of COMMD4 in GBM and LGG. CIBERSORT and TIMER were utilized to assess the associations between COMMD4 and immune cells. The Gene Set Enrichment Analysis (GSEA) was employed to examine the functional data. Furthermore, the link between COMMD4 expression and predicted treatment response was evaluated via CellMiner Cross-Database. Meanwhile, qRT-PCR was conducted to examine COMMD4 expression in human glioma. Finally, Migration and invasion of glioma cells (U-87, U-251) were assessed using transwell assays. R was used to analyze the statistical data.

**Results:** According to our findings, COMMD4 expression level was higher in patients having grade-dependent glioma who also showed an unfavorable prognosis. Furthermore, qRT-PCR confirmed the high expression of COMMD4 in glioma tissues and cells. Additionally, using integrated correlation analysis, we acquired significant prognostic findings between isocitrate dehydrogenase 1(IDH1) and COMMD4. Meanwhile, a link between COMMD4 and many tumor-infiltrating immune cells was observed. GSEA and drug response analysis revealed the potential mechanism of COMMD4 in drug resistance of glioma.

**Conclusion:** The current findings validated COMMD4 as a novel biological marker, which might offer insights into the possible drug resistance mechanisms and the impact of the immune microenvironment on glioma. COMMD4 might be used to predict glioma prognosis.

## 1 Introduction

Glioma is the most prevalent malignancy affecting the central nervous system (CNS), with roughly 4.7 cases per 100,000 persons being diagnosed each year (Larjavaara et al., 2007; Ostrom et al., 2013). Currently, the standard treatment plan for glioma is the combination of chemotherapy, radiotherapy, and surgical intervention. However, the prognosis remains unfavorable owing to a low sensitivity of glioma to radiotherapy and chemotherapy (Tonn et al., 2012; Jiang et al., 2016; Peng et al., 2018). Thus, a breakthrough in the treatment of glioma is critical. Nonetheless, the molecular mechanism in glioma is incompletely understood, hindering the development of novel treatment methods for glioma diagnosis and management (Lin et al., 2017 ).

In 2016, the World Health Organization (WHO) revised its categorization of CNS malignancies. According to the WHO classifications, adult diffuse gliomas are commonly identified and categorized by the nuclear retention, identification of the 1p/19q chromosomal co-deletion, and mutations in isocitrate dehydrogenase 1 (IDH1) or isocitrate dehydrogenase 2 (IDH2) genes. (Louis et al., 2014; Louis et al., 2016) In the 2021 revised version, novel molecular indicators, including telomerase reverse transcriptase (TERT) promoter alterations and epidermal growth factor receptor (EGFR) gene amplification, are required to classify adult patients with gliomas. Additional molecular indicators in gliomas include tumor protein p53 (TP53) mutation, which is related to poor prognosis and response to treatment (Louis et al., 2020; Louis et al., 2021).

The diffuse glioma encompasses both lower-grade gliomas (LGG) and glioblastomas (GBM), but a subgroup of tumors within each grade responds significantly differently to treatment. (Phillips et al., 2006) Even with such a heterogeneity, almost all glioma patients receive alkylating chemotherapy. The use of alkylators such as temozolomide (TMZ) could improve overall patient survival, but many patients experience only limited benefits. DNA repair enzyme O6-methylguanine DNA methyltransferase (MGMT) is thought to be the most effective mechanism of glioma resistance to TMZ. (Stupp et al., 2005; Hegi et al., 2008) Therefore, for the development of novel molecular targeting therapeutics, it is essential to identify tumor-specific pathways underlying DNA damage repair response initiation and hyperactivation.

The copper metabolism MURR1 domain (COMMD) protein family has ten members. COMMD proteins exert key roles in carcinogenesis, progression, invasion, and metastasis. (Green, 2003; Maine and Burstein, 2007; Wang et al., 2021) COMMD4 is a protein-coding gene belonging to the COMMD family, and is expressed at a high level in non-small cell lung cancers (NSCLC) and hepatocellular carcinoma (HCC). (Mao et al., 2011; Suraweera et al., 2020; Wang et al., 2021) Previous reports showed that in NSCLC cells, COMMD4 depletion results in apoptosis mediated by mitotic catastrophe, indicating that COMMD4 might serve as a therapeutic target. Nonetheless, it is unknown if COMMD4 could be employed as a biological marker for glioma and its involvement in gliomas is also unclear.

The data used in this research were obtained from the CGGA (Chinese Glioma Genome Atlas) and TCGA (The Cancer Genome Atlas) databases. Potential association between immune infiltration levels and COMMD4 in LGG and GBM was examined utilizing CIBERSORT. In addition, the Tumor Immune Estimation Resource (TIMER) was applied to evaluate the density of distinct Tumor-Infiltrating Immune Cells (TIICs). The link between COMMD4 expression and drug response was analyzed by CellMiner. This research improves the current understanding of the mechanisms and functions of COMMD4 in glioma.

## 2 Materials and methods

### 2.1 Retrieval and pre-processing of data from the cancer genome atlas

The LCG and GBM gene expression data and clinical data were extracted from the TCGA database (http://tcga-data. nci.nih.gov). The whole dataset had 698 tumors and 5 normal samples. (Neftel et al., 2019; Huang et al., 2021) Glioma sequencing data were generated utilizing the RNAseq - HTSeq platform and Strawberry Perl software (version 5.32.1). R (version 4.1.1) were used to conduct all the processing operations.

### 2.2 Clinical data and the CGGA mRNA matrix

The CGGA database (http://www.cgga.org.cn) is China’s most comprehensive glioma genome repository, which provided this study with 1319 glioma samples. Informed consent was obtained before the acquisition of all these samples. Premised on this information, we determined the variations and the survival values in COMMD4 expression. In addition, we obtained additional datasets including the mRNAseq_325 (Illumina HiSeq 2000 or 2500), mRNAseq_693 (Platform: Illumina HiSeq) and mRNA_array_301 (Agilent Whole Human Genome (array)) datasets. The mRNAseq_693 dataset contained 693 glioma samples, and the mRNAseq_325 dataset contained 325 glioma samples. After that, we employed the limma packages to normalize and batch the two mRNAseq matrices. Table 1 shows the clinicopathological parameters of patients whose clinical data from the CGGA database were complete. The survival and gene expression of COMMMD4 were listed in Tables 2, 3 using R software.

**TABLE 1:** Baseline of CGGA patients’ information.

		Total	Low expression	High expression	χ2	*p*
PRS_type	primary	502	253	249	1.0919	0.5793
	Recurrenrt	222	113	109		
	Secondary	25	10	15		
Grade	WHO II	218	143	75	36.6424	0
	WHO III	240	121	119		
	WHO IV	291	112	179		
Gender	Male	442	224	218	2.6105	0.1062
	Female	267	152	115		
Age	< =41	342	188	154	5.7294	0.017
	>41	407	188	219		
Radio_status	No	124	62	62	0.0024	0.961
	Yes	625	314	311		
Chemo_status	No	229	128	101	4.2792	0.0386
	Yes	520	248	272		
IDH_mutation_status	Wildtype	339	151	188	7.9289	0.004
	Mutant	410	225	185		
1p19q_codeletion_status	Non-codel	594	290	304	2.1825	0.1396
	Codel	155	86	69		

**TABLE 2:** Cox analysis of the CGGA database.

Id	HR	HR.95L	HR.95H	*p* Value
COMMD4	1.276706	1.144555	1.424117	<0.001
Histology	4.486991	3.695058	5.448654	<0.001
Grade	2.883411	2.526415	3.290853	<0.001
Gender	1.04351	0.865536	1.258081	0.655
Age	1.623833	1.345161	1.960236	<0.001
Radio	0.928909	0.719933	1.198546	0.571
Chemo	1.647389	1.327807	2.043888	<0.001
IDH_mutation	0.317158	0.262089	0.383798	<0.001
1p19q_codeletion	0.230575	0.169012	0.314561	<0.001

**TABLE 3:** Cox analysis of the TCGA database.

Characteristics	Total(N)	HR (95% CI)	*p* Value
COMMD4	695	2.238 (1.750–2.861)	<0.001
Histological type	695		
Astrocytoma	195	Reference	
Glioblastoma	168	6.791 (4.932–9.352)	<0.001
Oligoastrocytoma	134	0.657 (0.419–1.031)	0.068
Oligodendroglioma	198	0.580 (0.395–0.853)	0.006
WHO grade	634		
G2	223	Reference	
G3	243	2.999 (2.007–4.480)	<0.001
G4	168	18.615 (12.460–27.812)	<0.001
Gender	695	1.262 (0.988–1.610)	0.062
Age	695	4.668 (3.598–6.056)	<0.001
IDH status	685	0.117 (0.090–0.152)	<0.001
1p/19q codeletion	688	4.428 (2.885–6.799)	<0.001

### 2.3 Interaction analysis of gene expression profiles

GEPIA (http://gepia.cancer-pku.cn/) is an online interactive server that comprises 8587 normal clinical specimens and the RNA seq data of 9736 tumors acquired from TCGA and The Genotype-Tissue Expression (GTEx) datasets. GEPIA was utilized here to investigate the clinical functions of COMMD4. (Tang et al., 2017) The bipartite method was applied to classify the COMMD4 expression into high- and low-expression groups. In addition, the “survival” modules were utilized to examine the links between COMMD4 expression and glioma patients’ prognosis. Furthermore, the variation in the expression levels of COMMD4 between the tumor and normal samples was evaluated by the boxplot modules with the disease status as variables (normal or tumor). We employed the Wilcoxon rank-sum test to examine the links between COMMD4 expression and grade, 1p/19q codeletion status, and IDH mutation status. The R software was used with the tools such as survminer, survival, and ggplot.

### 2.4 Univariate cox analysis

The links between histology, grade, 1p/19q-codeletion status, IDH mutations, and COMMMD4 expression were analyzed by the Univariate Cox analysis. We performed a statistical study using data from the CGGA and TCGA databases with the survival function in R (version 4.1.1).

### 2.5 Gene set enrichment analysis analysis

GSEA including KEGG and GO analyses was employed to examine the functional enrichment of COMMMD4 expression. The biological coherence and correlations among each predicted module were investigated using GO analysis with differentially expressed mRNAs in the GO categories. To explore key pathways linked with COMMMD4 expression, KEGG analysis was carried out.

### 2.6 Immune cell infiltration assessment

Associations of TIICs with gene expression profiles in tumor tissues were assessed with the ssGSEA and CIBERSORT algorithms. The ssGSEA technique was used to calculate the relative infiltration levels of 24 distinct immune cells in the TCGA dataset. The “ggplot2” software was used to visualize the calculated Spearman correlations of 24 distinct immune cell infiltrations with hub genes. In cell type development, the CIBERSORT method employs a vector regression model. The consistent performance of CIBERSORT could be used to evaluate cellular heterogeneity on gene expression profiles of complex tissues. The algorism was then introduced to transfer the standard-annotated gene expression data to the CIBERSORT website after being applied to the LM22-signed matrix (Lin et al., 2021; Sun et al., 2022; Zhang et al., 2022). The data obtained were classified into low- and high-COMMMD4 expression subgroups in order to examine the variations in the percentage of immune cells, including macrophages, T cells, monocytes, NK cells, dendritic cells, and B cells.

### 2.7 Tumor immune estimation resource database analysis

Tumor Immune Estimation Resource (TIMER) (https://cistrome.sh.inyapps.io/timer/) was utilized to visualize the correlations between the series of variables in 32 kinds of cancers and over 1000 TCGA samples and immune infiltration levels. (Li et al., 2017) TIMER uses a deconvolutional statistical approach to produce an inference on multiple TIICs. Gene modules were employed to examine the connection between COMMD4 expression levels and TIICs, which included CD8^+^ T cells, B cells, macrophages, neutrophils, dendritic cells, and CD4^+^ T cells. The log2 TPM was applied to show the level of gene expression.

### 2.8 Single-cell analysis

Tabula Muris ( https://tabula-muris.ds.czbiohub.org/) is a single-cell transcriptome tool containing over 100,000 cells from 20 different tissues and organs. (Tabula Muris Consortium et al., 2018) Using this database, we examined the associations of COMMD4 expression levels with various types of cells and tissues, including endothelial cells and T lymphocytes. Fluorescence-activated cell sorting (FACS) was also employed here to analyze the connections between COMMMD4 expression and distinct types of cells with great sensitivity and coverage.

### 2.9 COMMD4 and drug response

A link between COMMD4 expression and drug responsiveness was established by CellMiner (http://discover.nci.nih.gov/cellminer/). CellMiner, which was created by the Genomic and NCI, CCR, DTB, Pharmacology Facilit, NIH, is a query tool and database created for cancer researchers to facilitate the incorporation and evaluation of molecular as well as pharmacologic data for the NCI-60 tumor cell lines. The NCI-60 is a panel comprising 60 distinct human tumor cell lines, and is utilized by the National Cancer Institute’s Developmental Therapeutics Program to identify more than 100,000 chemical compounds and natural products (Shankavaram et al., 2009).

### 2.10 Quantitative RT-PCR

Total RNA was extracted from paraneoplastic tissue and tumor tissue from glioma patients of different grades using the TRIzol reagent (Sigma-Aldrich, United States). Cell line samples were processed in the same way. Then, RNA from each sample (2 μg) was reverse-transcribed into cDNA, after which reverse transcription-quantitative polymerase chain reaction (RT-qPCR) was performed using the FastStart universal SYBR ^®^Green Master (Roche, United States) in an ABI QuantStudio5 Q5 real-time PCR System (Thermo Fisher Scientific, United States). The template for the reaction was selected as cDNA at a reaction volume of 20 μl (10 μl of PCR mixture, 0.5 μl reverse and forward primers, 2 μl of cDNA template, and an appropriate volume of water). For the PCR reactions, the cycling conditions began with DNA denaturation at 95°C for 30 s (s), followed by 45 cycles for 15 s at 94°C, 30 s at 56°C, and 20 s at 72°C. Each sample was performed in triplicates. The 2^−ΔΔCT^ method was adopted to obtain threshold cycle (CT) measurements, which were standardized to glyceraldehyde 3-phosphate dehydrogenase (GAPDH) levels in all samples. The mRNA expression levels were compared to paracancerous tissue controls. The following are the sequences of primer pairs for the target genes:

**Table:** 

**Gene**	**Forward primer sequence (5–3)**	**Reverse primer sequence (5–3)**
COMMD4	TTCTTGGCGCGATGAGGTTC	TCAGAGGGCGTGACTCCATA
GAPDH	AATGGGCAGCCGTTAGGAAA	GCCCAATACGACCAAATCAGAG

### 2.11 Cell culture and drug

Human glioma cell lines U-87 and U-251 were obtained from ATCC (Beijing Beina Chuanglian Biotechnology Institute) and cultured in F12 and DMEM containing 10% fetal bovine serum (Gibco, Carlsbad, CA, United States), respectively. Both cell lines were stored in a humidified incubator at 37°C with 5% CO2. Temozolomide was procured from MCE (CAT# HY-17364). Dissolution of temozolomide was carried out in dimethyl sulfoxide (DMSO, Beyotime). Finally, it was co-cultured with cells at a concentration of 20 µM/ml.

### 2.12 Transwell assay

Transwell assays for migration and invasion of glioma cells (U-87, U-251) were performed. Briefly, cells (5 × 104) were inoculated into chambers coated (for invasion) or uncoated with Matrigel (BD Biosciences, San Jose, CA) (for migration). Serum-free medium was added to the upper layer and a complete DMEM medium was added to the lower layer. After 24 h of incubation, migrating or invading cells were fixed with 4% paraformaldehyde and stained with 0.1% crystalline violet. Counting under a light microscope.

## 3 Results

### 3.1 Relationship between COMMD4 expression and glioma survival status

**FIGURE 1:**
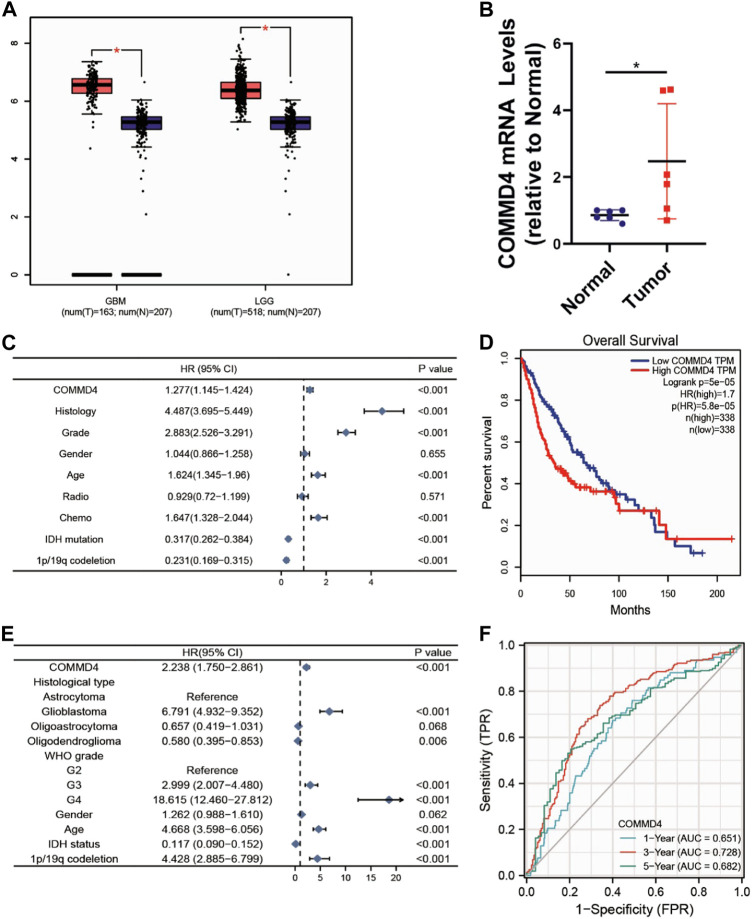
**(A)** COMMD4 expression differs significantly between GBM and LGG. **(B)** qRT-PCR assays to measure the mRNA expression level of COMMD4 in paraneoplastic tissue and tumor tissue from glioma patients. (*p < 0.05, with student’s t-test). COMMD4 expression and other clinicopathological parameters derived from **(C)** CGGA dataset and **(E)** TCGA datasets were subjected to a univariate Cox analysis. **(D)** GEPIA was used to assess the survival curves of various COMMD4 expression levels. **(F)** The time-dependent receiver operating characteristic (ROC) curves for survival rates over one, three, and 5 years.

COMMD4 expression level was elevated in both GBM (num (N) = 207, num (T) = 163) and in LGG (num (N) = 207 num, (T) = 518; Figure 1A). Furthermore, the COMMD4 overexpression was indicative of a more unfavorable overall survival (OS) (num (high) = 338, num (low) = 338, *p* < 0.001; Figure 1D). By using the bipartite technique, the expression level of COMMD4 in normal and malignant tissues was classified into 2 groups (low- and high-expression groups). These findings demonstrated that COMMD4 expression levels were greater in tumor tissues and were linked to a worse OS.

### 3.2 COMMD4 as an independent predictor for glioma patients

Based on the CGGA and TCGA databases, univariate Cox analysis was conducted to assess the utility or practicality of COMMD4 expression. Factors such as COMMD4 expression (*p* < 0.001), histology (astrocytoma, oligodendroglioma, Glioblastoma) (*p* < 0.05), grade (WHO grade) (*p* < 0.001), chemotherapy (*p* < 0.001), IDH mutation (*p* < 0.001) and 1p19qcodeletion (*p* < 0.001) (Figures 1C,E) were determined premised on the univariate analysis. According to the receiver operating characteristic (ROC) analysis, the area under the curve (AUC) of COMMD4 was found to be 0.651, 0.728, and 0.682 for one-, three-, and 5-year survival, respectively (Figure 1F).

### 3.3 The relationships between COMMD4 expression and world health organization grade, isocitrate dehydrogenase 1 phenotype in the chinese glioma atlas and the cancer genome atlas

**FIGURE 2:**
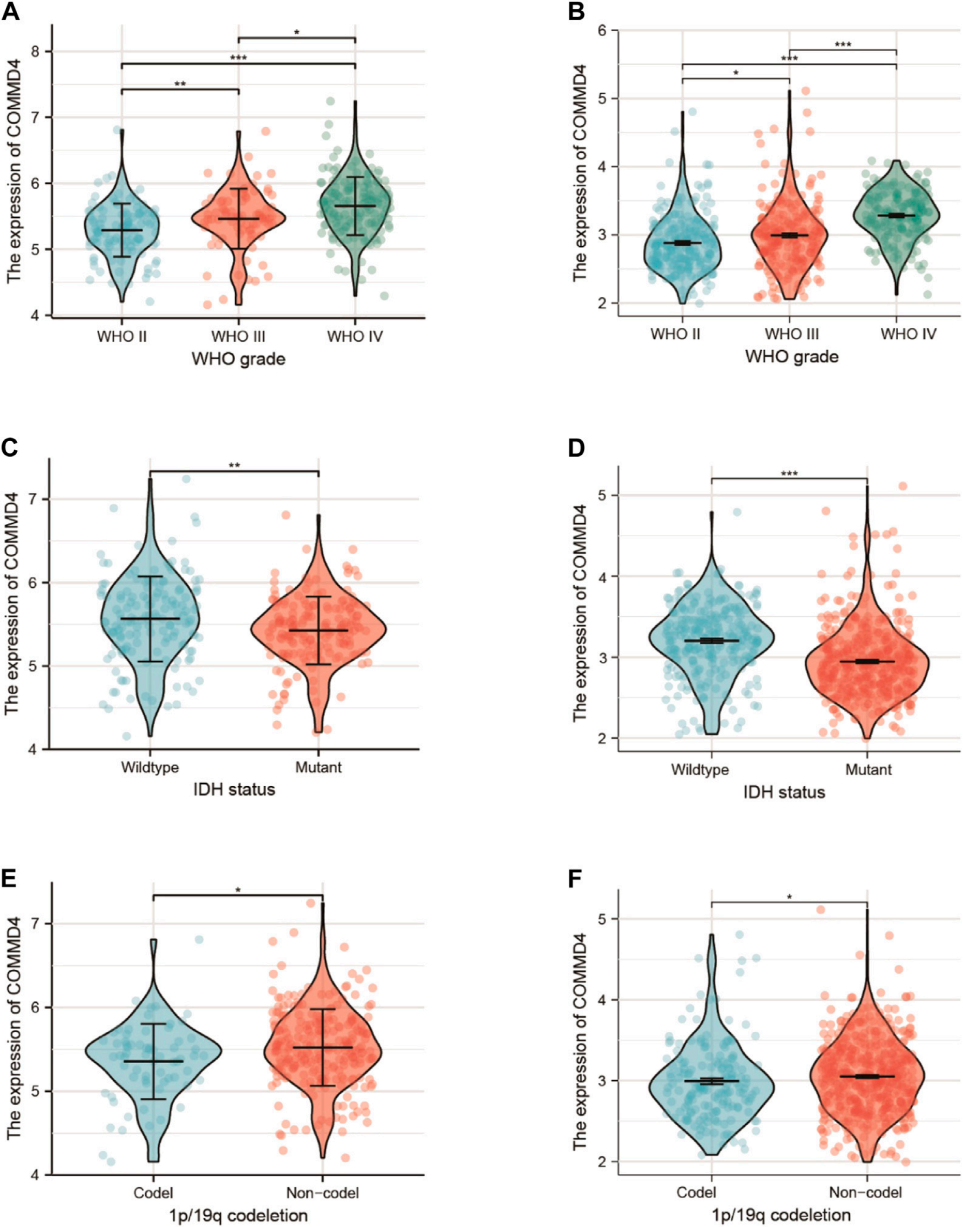
Expression of COMMD4 in CGGA **(A)** WHO grades. **(C)** IDH status-stratified distribution. **(E)** 1p/19q-codeletion status distribution. COMMD4 expression in TCGA **(B)** WHO grades. **(D)** IDH status-stratified distribution. **(F)** 1p/19q-codeletion status distribution.

The relationships between COMMD4 expression, WHO grade, and IDH1 state were analyzed in the two different datasets. In both datasets, comparable associations between COMMD4 expression levels and WHO glioma grades could be found (Figures 2A,B). The elevated COMMD4 expression level was linked to greater glioma malignancy, according to the findings. Furthermore, the IDH-wildtype group showed substantially elevated COMMD4 expression level compared with that in the IDH-mutant subgroup (Figures 2C,D). The 1p/19q-non-codeletion (non-codel) group had a considerably elevated COMMD4 expression level compared with that of the 1p/19q-codeletion group (Figures 2E,F), which was calculated using the Wilcoxon rank-sum test. These findings illustrated that COMMD4 was expressed at a high level in the 1p19q-non-codeletion and IDH-wildtype groups.

### 3.4 Survival analysis and expression of COMMD4 in primary gliomas derived from the chinese glioma atlas database

**FIGURE 3:**
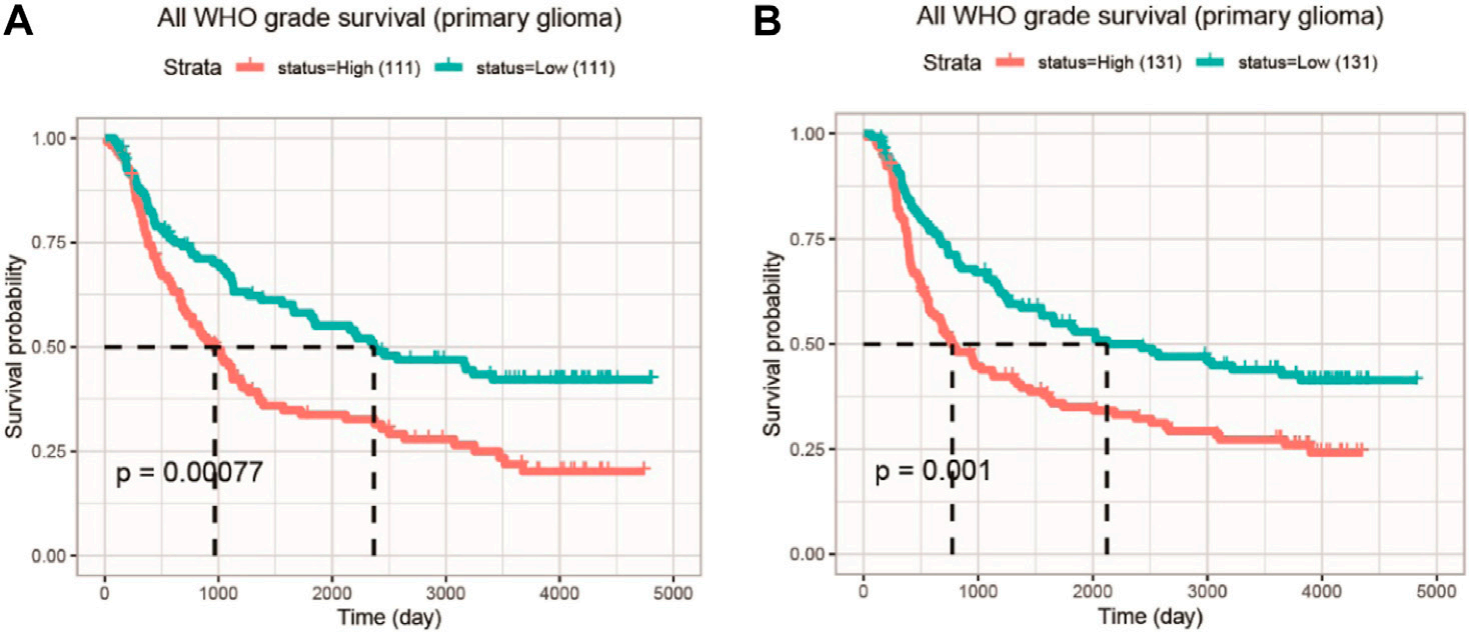
The KM survival curve illustrating the expression of COMMD4 in GBM and LGG patients **(A)** Dataset ID: mRNAseq_325-Primary Glioma **(B)** Dataset ID: mRNA_array_301-Primary Glioma.

With the two CGGA datasets, an integrative survival analysis was performed to examine the association of COMMD4 expression with survival of glioma patients. In Dataset 1 (ID: mRNAseq 325), patients in the high-COMMD4 expression group with primary glioma demonstrated an unfavorable prognosis (*p* < 0.001; Figure 3A). Furthermore, the high-expression group in Dataset 2 (ID: mRNA array 301) had a significantly unfavorable prognosis in primary glioma (*p* = 0.001; Figure 3B).

### 3.5 Multifactorial integrated survival analysis in the chinese glioma atlas database

**FIGURE 4:**
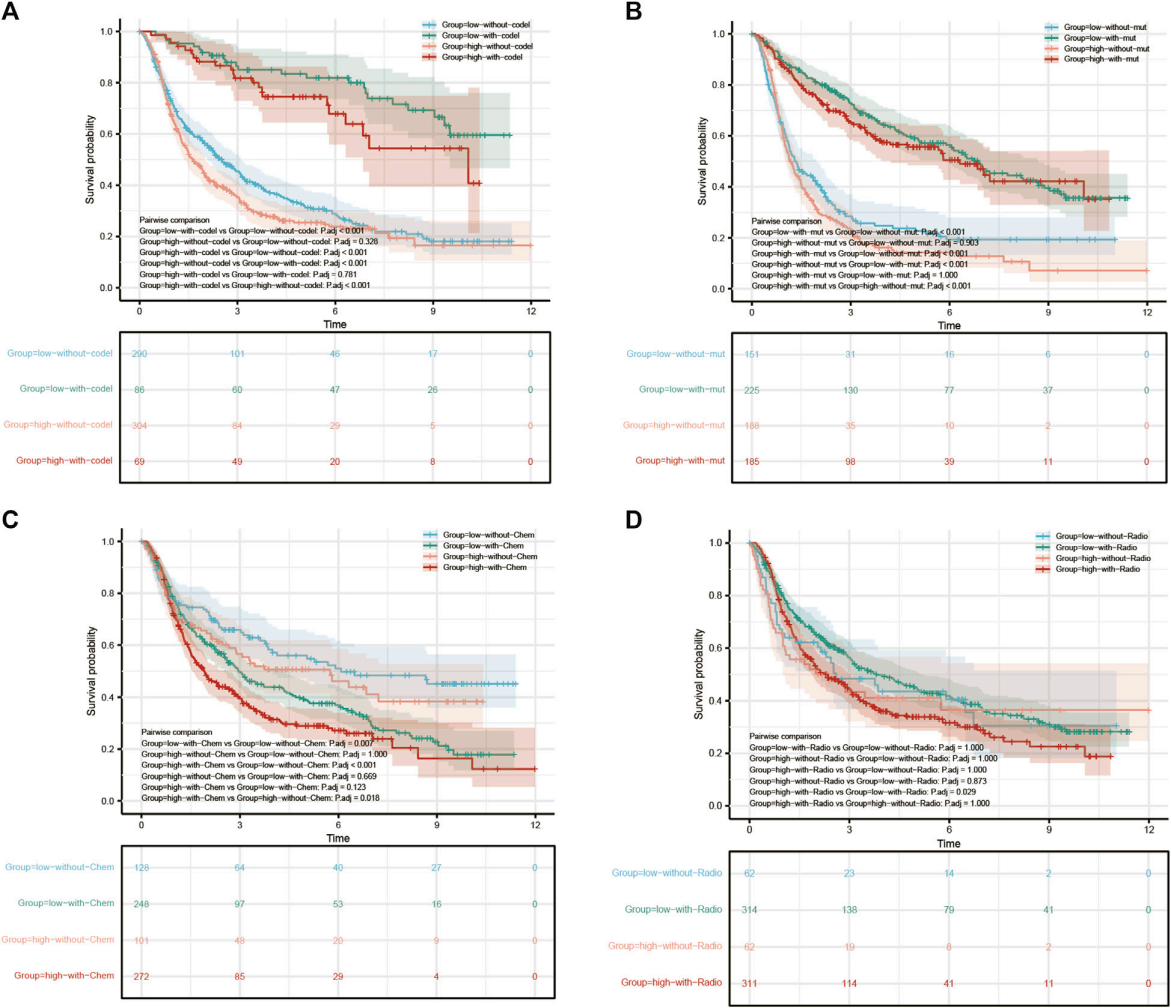
Survival analysis of GBM and LGG patients with varying levels of COMMD4 expression in comparison with **(A)** 1p/19q status **(B)** IDH mutation **(C)** chemotherapy **(D)** radiotherapy.

To further analyze the clinical relevance of COMMD4, 1p19q status (Figure 4A), IDH1 genotypes (Figure 4B), chemotherapy (Figure 4C), radiotherapy (Figure 4D) were incorporated as parameters in a multivariate analysis. As demonstrated by the 1p19q status, COMMD4 overexpression and 1p19q non-codeletion (orange in Figure 4A) were associated with the poorest prognosis. Notwithstanding a high expression level of COMMD4, the survival rate remained high in the IDH1-R132-mutant groups (red Figure 4B). Thus, COMMD4 could be seen as a viable marker in the corresponding IDH1 genotypes (*p* < 0.0001). Following that, we examined the link between COMMD4 expression and the survival of patients receiving chemotherapy, and the worst prognosis was found in the high-COMMD4 expression group after chemotherapy (red in Figure 4C). However, favorable prognoses were reported in patients in the low-COMMD4 expression group who did not receive chemotherapy (blue in Figure 4C). As a result, patients receiving chemotherapy with a low COMMD4 expression level may benefit more. Similarly, patients in the high-COMMD4 expression group receiving radiotherapy (red in Figure 4D) were found to have unfavorable prognosis in comparison to those in the low-expression group with radiotherapy (green in Figure 4D).

### 3.6 Gene set enrichment analysis investigation of COMMD4-related pathways

**FIGURE 5:**
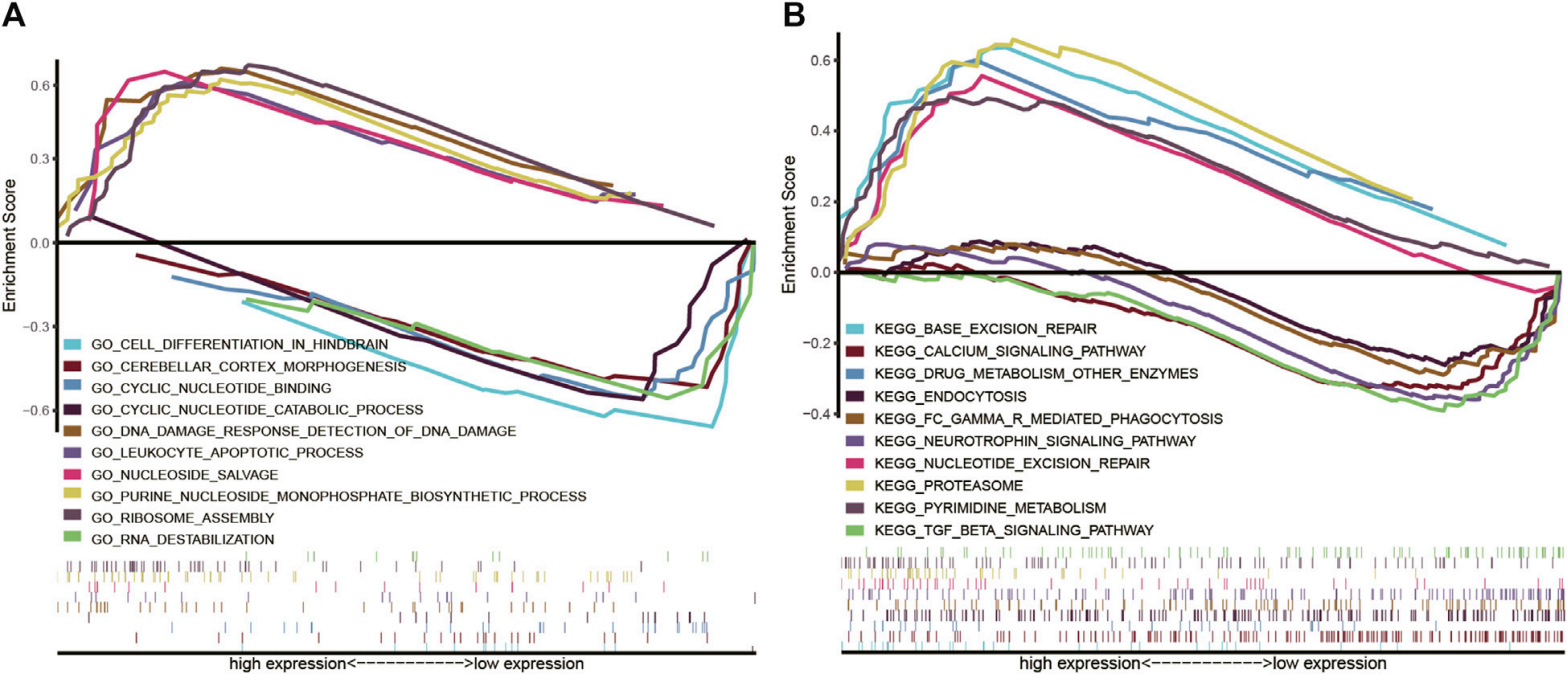
**(A)** GO findings show the pathways that were associated with COMMD4. **(B)** KEGG analyses showed the pathways that were linked to COMMD4.

We performed GO and KEGG analysis to examine the potential biological role of COMMD4. We identified five gene pathways strongly linked to COMMD4 expression, and discovered that COMMD4 was remarkably related to repair-related and immune-related gene pathways. According to the findings of GO analysis, the five pathways closely associated with the elevated level of COMMD4 overexpression included DNA damage response detection, leukocyte apoptotic process, nucleoside salvage, purine nucleoside monophosphate biosynthetic process, and ribosome assembly. Additionally, five inversely correlated categories were found, including cell differentiation in the hindbrain, cyclic nucleotide-binding, RNA destabilization, cerebellar cortex morphogenesis, and cyclic nucleotide catabolic process (Figure 5A). The findings of KEGG analysis indicated that the five pathways were positively linked to upregulation of COMMD4 expression, including base excision repair, drug metabolism of other enzymes, nucleotide excision repair, proteasome, and pyrimidine metabolism. Similarly, the five categories inversely linked to COMMD4 expression upregulation were FC gamma r mediated phagocytosis, calcium signaling pathway, endocytosis, TGF beta signaling pathway, and neurotrophin signaling pathway (Figure 5B).

### 3.7 Associations between COMMD4 expression and tumor-infiltrating immune cells

**FIGURE 6:**
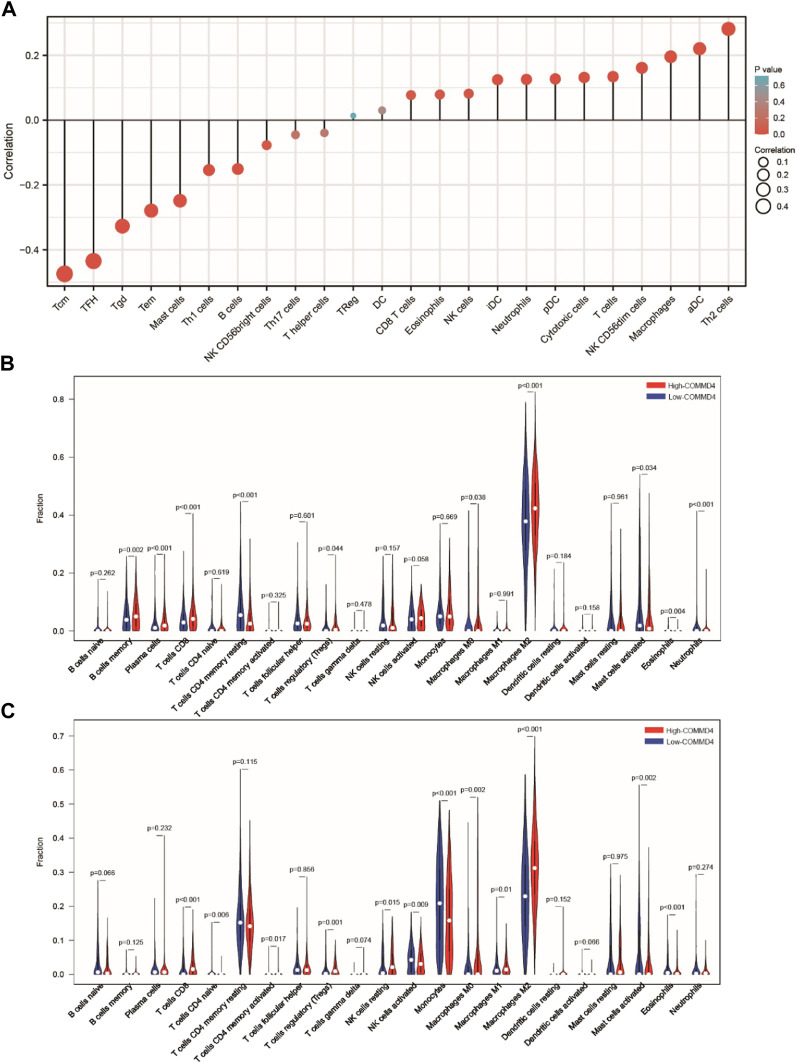
**(A)** The findings of the relative ratios of TIIC computed utilizing the ssGSEA method premised on the TCGA dataset. The relative ratios of TIIC derived utilizing the CIBERSORT method premised on the **(B)** CGGA and **(C)** TCGA datasets.

The relationship between TIICs in glioma and COMM4 expression levels was investigated. According to our results, the COMMD4 expression had a negative correlation with Tgd, TFH, Tem, Tcm, Th1 cells, Th2 cells, and Mast cells. (Figure 6A). To further verify the relationship between TIICs in glioma and COMMD4, the 703 TCGA samples and the 1018 CGGA samples were separated into low- and high- COMMD4 expression groups. According to the samples from the CGGA database, immune cell infiltration level (activated Mast cells, resting memory CD4^+^ T cells, activated memory CD4^+^ T cells, Neutrophils) was shown to be considerably lower in the high-risk group than the low-risk group. Furthermore, the infiltration levels of immune cells (activated Mast cells, naive CD4 T cells, activated NK cells, Monocytes, and naïve CD4 T cells) were considerably lowered in the high-risk group than the low-risk group. In both the CGGA (Figure 6B) and the TCGA (Figure 6C) databases, Mast cell activation (*p* < 0.05) was greatly attenuated in the high-COMMD4 expression subgroup.

### 3.8 COMMD4 expression was related to the infiltration levels of immune cells and overall survival in glioblastomas and lower-grade gliomas from tumor immune estimation resource

**FIGURE 7:**
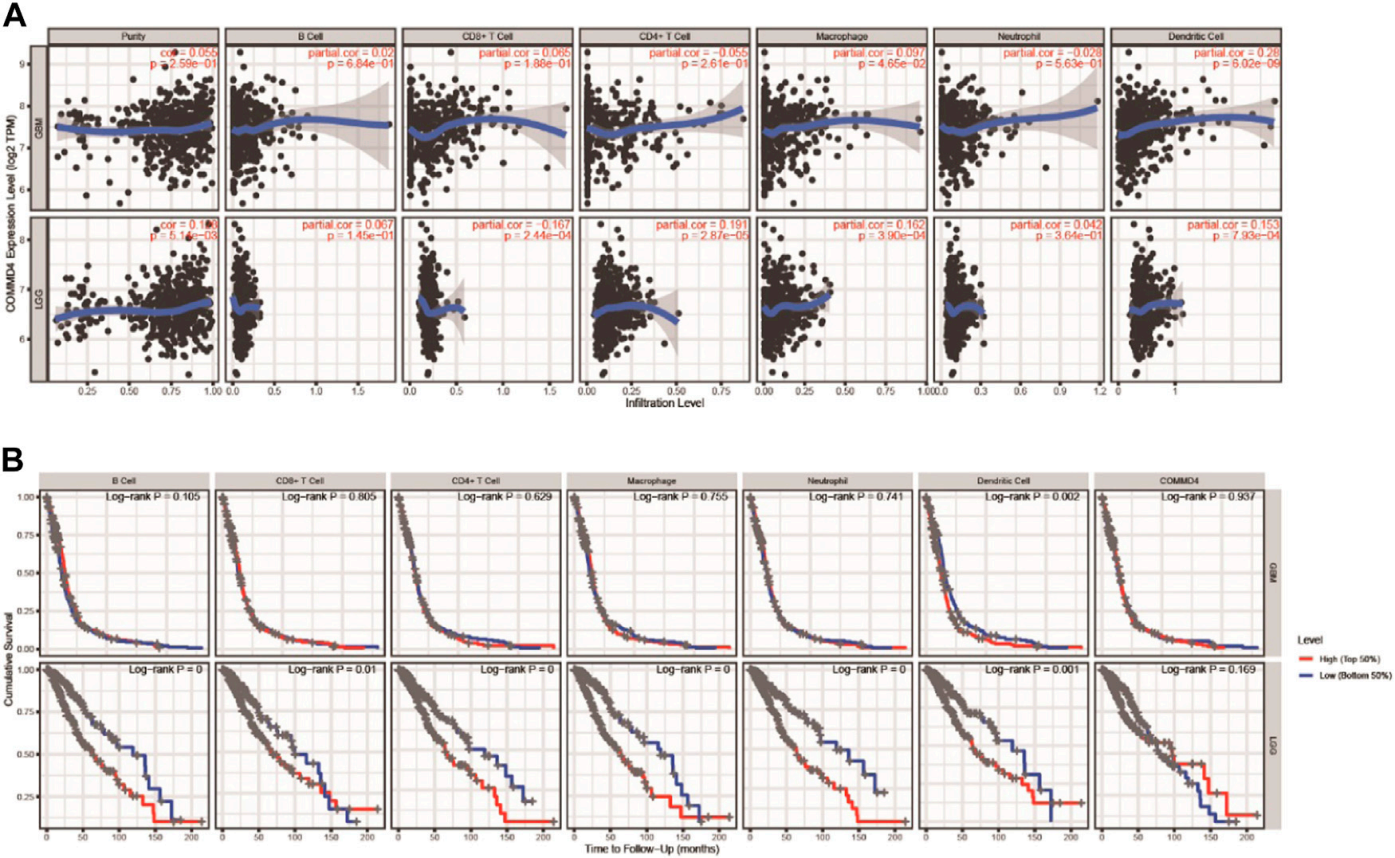
**(A)** In GBM and LGG, COMMD4 expression levels have substantial associations with infiltration levels of B cells, T cells, Macrophages, Neutrophils, and DCs. **(B)** B cells, T cells, Macrophages, Neutrophils, and DCs are all associated with overall survival in patients with GBM and LGG.

The TIMER database was used to investigate whether the immune infiltration levels in glioma were linked to the COMMD4 expression levels. The infiltration levels of CD8^+^ T lymphocytes was inversely linked to the expression of COMMD4 (r = −0.167, *p* = 2.44e-04) (Figure 7A) in LGG. Furthermore, the factors of neutrophils, DCs, macrophages, T and B cells were related to the OS rate in LGG and GBM (Figure 7B).

### 3.9 COMMD4 expression and cells from various organs were examined by single-cell analysis

**FIGURE 8:**
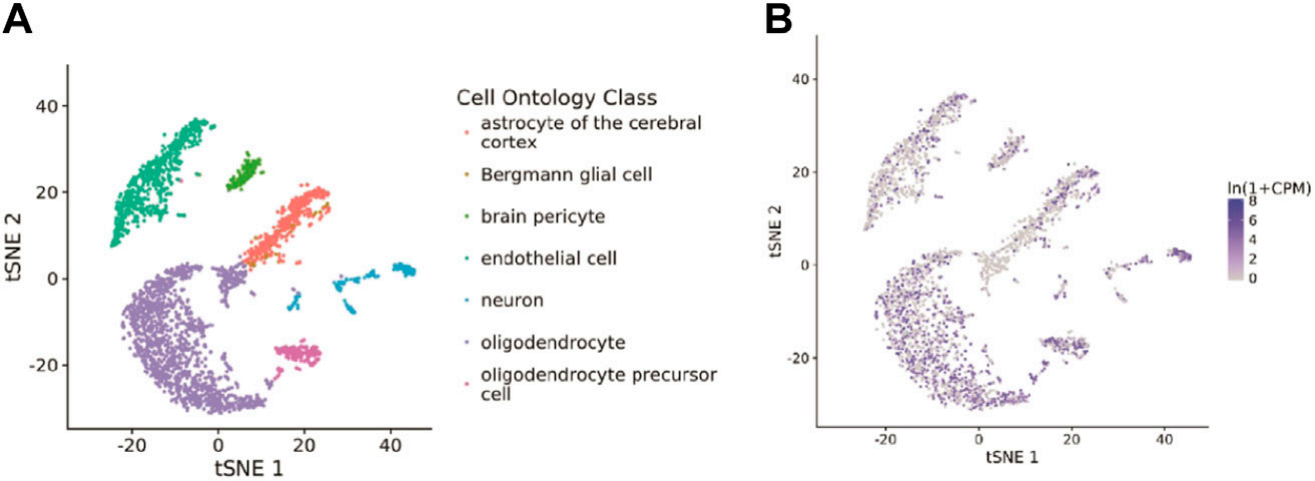
Single-cell analysis of COMMD4 expression **(A)** The cells that were linked to the tissues extracted from the brain. **(B)** The COMMD4 expression in tissues extracted from the brain.

The Tabula Muris database was used to examine the associations between COMMD4 expression and cells. Glioma were closely associated with astrocytes of the brain pericyte, neuron, oligodendrocyte precursor cell, oligodendrocyte, endothelial cell, and Bergmann glial cell, as shown in Figure 8A, and were displayed using t-SNE from FACS cells. As depicted in Figure 8B, COMMD4 was primarily associated with oligodendrocytes.

### 3.10 COMMD4 and drug responsiveness

**FIGURE 9:**
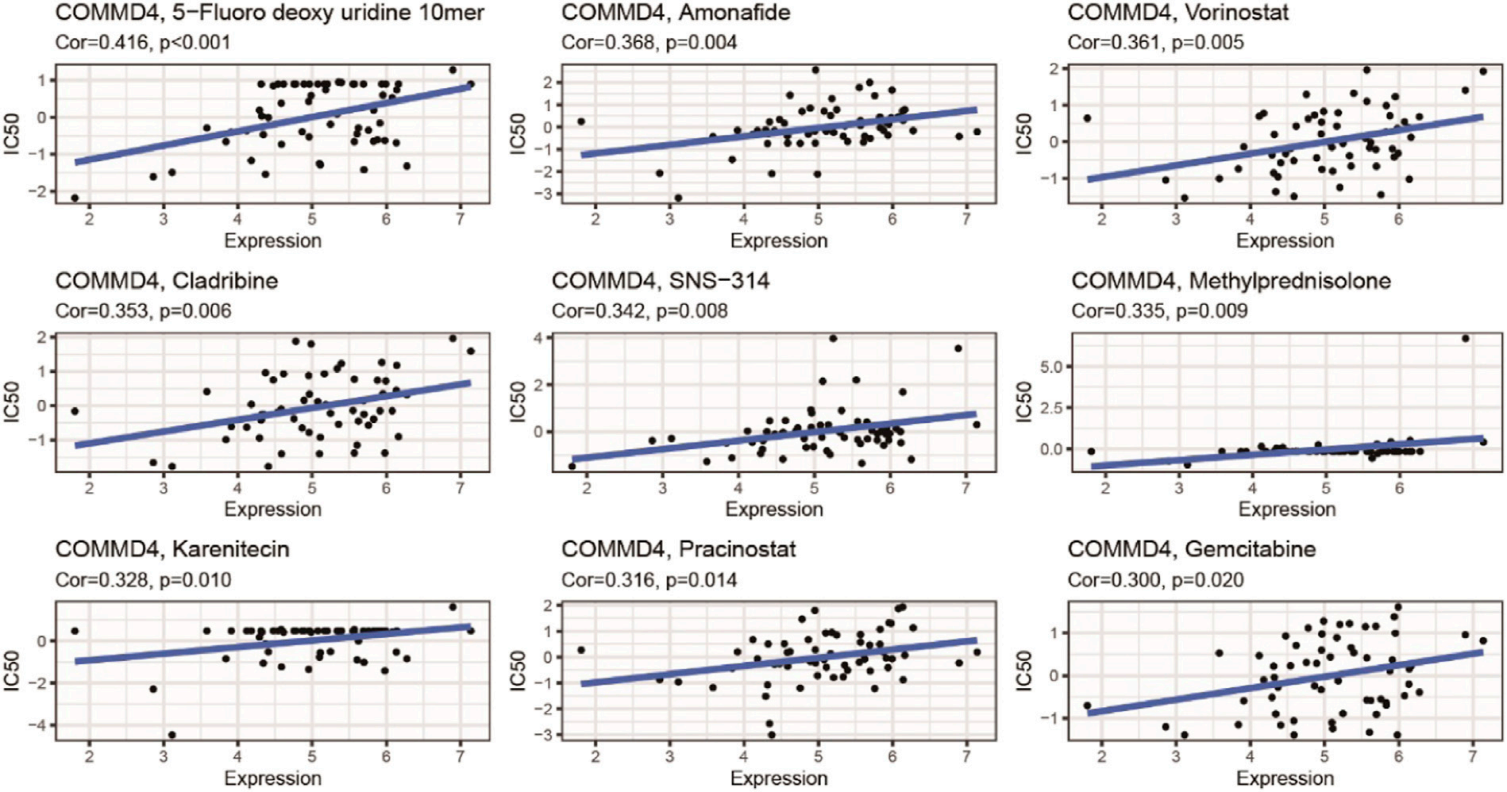
An illustration of the relationship between COMMD4 expression and expected medication response.

COMMD4 expression was inversely related to drug responsiveness among patients treated with 5−Fluoro deoxy uridine, Amonafide, Vorinostat, Cladribine, Triethylenemelar, Hydroxyurea, Thiotepa, SNS−314, Methylprednisolone, Karenitecin, Pracinostat, and Gemcitabine. Figure 9 depicts the association between COMMD4 expression and predicted drug responsiveness.

### 3.11 The COMMD4 expression in human glioma

**FIGURE 10:**
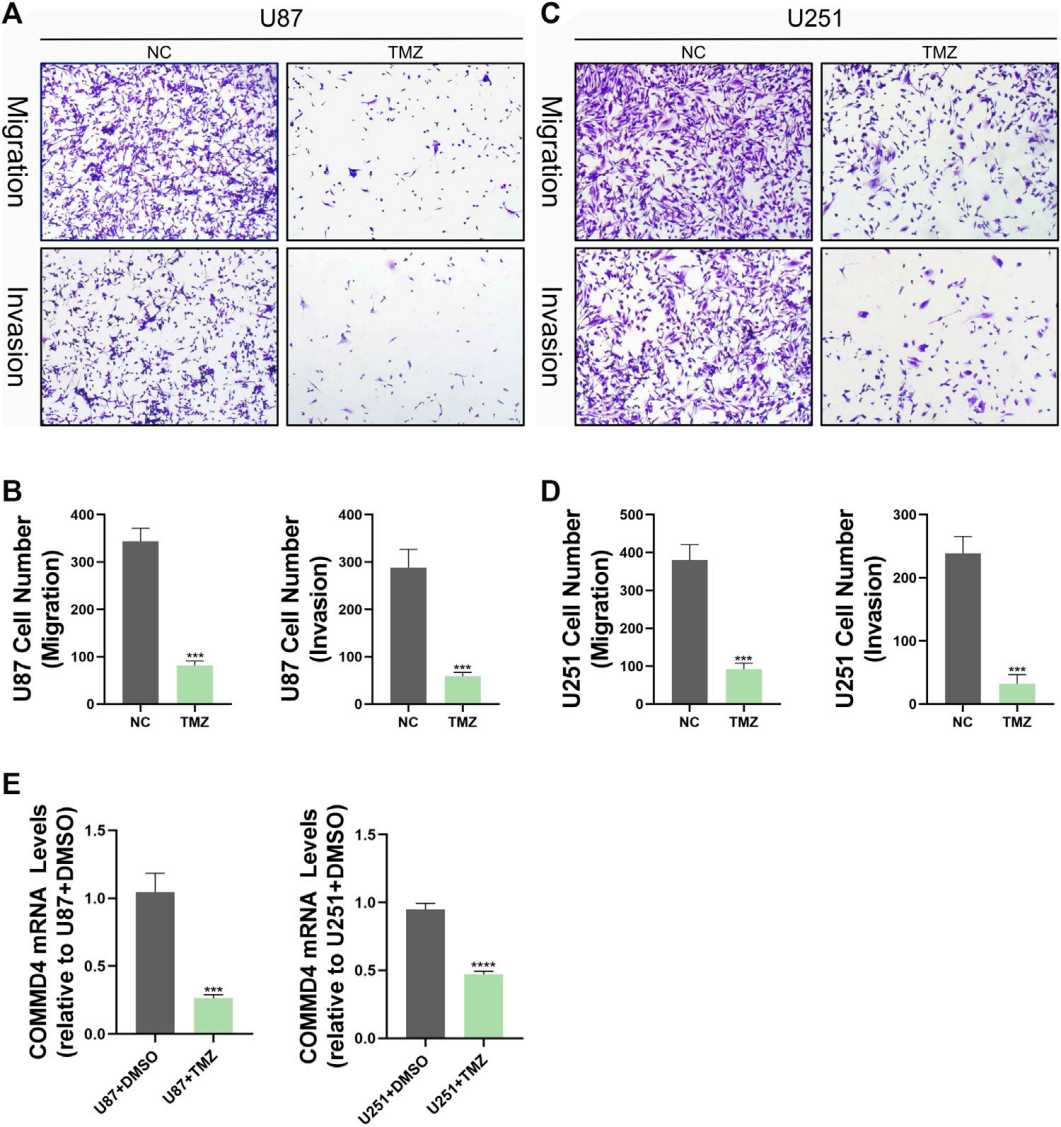
TMZ inhibits migration and invasion of glioma cells *in vitro* and reduces COMMD4 expression. **(A–D)** Transwell assay images of migration and invasion in the NC and TMZ groups, and quantitative counts of cell numbers **(E)** Relative quantitative analysis of COMMD4 expression in the NC and TMZ groups.

The level of COMMD4 expression in paraneoplastic tissue and tumor tissue from glioma patients was initially examined in this research. According to the RT-qPCR data, the expression of COMMD4 was up-regulated in glioma tissues relative to adjoining tissues (Figure 1B). Furthermore, glioma cells invasion and migratory abilities were evaluated by using transwell assay results in Figures 10A–D exhibited that the abilities of invasion and migration of U87 and U125 cells were conspicuously reduced by TMZ as comparison to the control group. In addition, results from qRT-PCR indicated the expression of COMMD4 was significantly upregulated after the induction of TMZ in these two cell lines (Figure 10E).

## 4 Discussion

Glioma is a type of brain tumor that originates from glial cells in the CNS and accounts for over 80% of all malignancies occurring in the brain. (Chen et al., 2017; Zhong et al., 2019)Surgical intervention and postoperative enhanced radiotherapy and chemotherapy are widely implemented in glioma treatment. (Bush et al., 2017; Malta et al., 2018) Glioblastoma, on the other hand, has an unfavorable prognosis, with a median survival period of shorter than 2 years. Therefore, viable biomarkers for early glioma detection are beneficial to patient management and prognosis.

This investigation proved the significance of COMMD4 in the pathogenesis of glioma, identified a new possible therapeutic target for glioma treatment and a prognostic indicator. In LGGs and GBMs, patients diagnosed with glioma exhibited low survival rate and elevated COMMD4 expression level. This study also examined the relationships between COMMD4 expression and IDH1 status. IDH1 phenotypes, according to the WHO, are an innovative diagnostic technique employed in clinical settings, and IDH1 mutation status is utilized to classify diffuse glioma in adults. The elevated expression levels of COMMD4 accelerated the malignant progression of glioma, as evidenced by the IDH1-wildtype patients’ unfavorable survival. Furthermore, we compared chemotherapy and radiotherapy to highlight the role of COMMD4 and identified COMMD4 as a molecular marker for glioma patients’ prognosis.

Although the mechanisms of COMMD4 in glioma cells are unknown, various research reports have shown that it is intimately linked to tumor genomic stability and apoptosis. Suraweera et al. found that COMMD4 was subjected to overexpression in NSCLC cells, and that siRNA knockdown of COMMD4 attenuated cell proliferation and viability. After being exposed to DNA-damaging agents, cell death was more accelerated. Following COMMD4 knockdown, non-small cell lung cancer (NSCLC) cells experienced mitotic catastrophe and apoptosis. Meanwhile, higher expression of COMMD4 has been found in NSCLC and was linked to unfavorable prognosis in adenocarcinoma (ADC). In addition, a previous report illustrated that COMMD4 maintains genomic integrity through regulating chromatin structure at DSB sites. Moreover, the researchers also discovered that cells lacking COMMD4 are more susceptible to multiple DNA-damaging agents that induce DSBs and are less effective in repairing DSBs. Though the association between COMMD4 and glioma was not yet completely clarified, it could be speculated that COMMD4 influenced the development of pathophysiological pathways of glioma based on our findings and previous research on COMMD4 (Suraweera et al., 2020; Suraweera et al., 2021).

GSEA was used to conduct GO terms and KEGG pathway analyses to further examine the possible biological roles of COMMD4 in glioma. In samples exhibiting low and high levels of COMMD4, GSEA indicated substantial differences in GO term and KEGG pathway enrichment. In particular, GESA analysis illustrated an enrichment of several immune-related and repair-related gene sets in the high-COMMD4 group, including leukocyte apoptotic process, DNA damage response detection of DNA damage, nucleoside salvage, and nucleotide excision repair. Notably, according to a growing body of research, DNA damage repair and immunological infiltration are both implicated in cancer advancement and drug resistance. These data indicated that COMMD4 was implicated in the progression of glioma. In glioma development, high COMMD4 expression level might affect mechanisms of treatment resistance and tumor immunology. Our findings suggested that the upregulation of COMMD4 expression was linked to a poor prognosis. We hypothesized that elevated COMMD4 expression level had a pivotal regulatory function in these oncogenic pathways, and this resulted in a poorer prognosis for glioma patients.

From CellMiner, we discovered that COMMD4 expression was adversely related to drug responsiveness in patients treated with Amonafide and Cladribinethe. The drug resistance of Amonafide and Cladribinethe may be related to the DNA damage repair function of COMMD4 (De Isabella et al., 1995; Liu et al., 2011). Furthermore, GSEA confirmed the substantial enrichment of immune-related gene sets in the high-COMMD4 expression group. We then examined the relationship between infiltration levels of immune cells in glioma and COMMD4 expression. COMMD4 expression demonstrated a strong negative association with the infiltration level of mast cells (MC), according to CIBERSORT analysis. Mast cells are specific immune system cells that release a wide range of physiologically active chemicals, which can activate, regulate, or decrease the immune response. (Gordon and Galli, 1990; Falduto et al., 2022; Fereydouni et al., 2022) When exposed to FcϵRI, Human MCs produce substantial levels of granulocyte-macrophage colony-stimulating factor (GM-CSF), according to Fereydouni et al. This is significant because both GM-CSF and TNF-α have been shown to attenuate tumor cell proliferation, promote tumor regression, and improve anti-tumor co-therapies. (Yan et al., 2017; Josephs et al., 2018; Plotkin et al., 2019) Our findings suggested that the negative impact of COMMD4 on glioma could be resulted from the reduced density of mast cells. We speculated that COMMD4 may have certain effects on tumor immunity.

In summary, this is the first research exploring the function of COMMD4 in glioma. COMMD4 level was elevated in gliomas and COMMD4 was associated with tumor grade. In addition, qRT-PCR verified the high expression of COMMD4 in glioma tissues and cells. Furthermore, a high level of COMMD4 overexpression was related to an unfavorable prognosis and impaired infiltration of immune cells in glioma. Finally, the primary glioma pathway mediated by COMMD4 may be connected to genomic stability, which may be associated with glioma treatment resistance. The study also had certain limitations, for instance, there was an absence of *in vitro* and *in vivo* trials. Thus, additional research was encouraged to identify COMMD4 as a viable prognostic marker in glioma treatment resistance.

## Data Availability

The original contributions presented in the study are included in the article/Supplementary Material; further inquiries can be directed to the corresponding authors.
